# Global research on NK cells in miscarriage: a bibliometric study

**DOI:** 10.3389/fmed.2025.1513213

**Published:** 2025-02-17

**Authors:** Yinan Wang, Xiaoqin He, Chaogang Yang, Jinli Ding

**Affiliations:** ^1^Reproductive Medical Center, Renmin Hospital of Wuhan University and Hubei Clinic Research Center for Assisted Reproductive Technology and Embryonic Development, Wuhan, China; ^2^Teaching Office, Renmin Hospital of Wuhan University, Wuhan, China; ^3^Department of Gastrointestinal Surgery, Zhongnan Hospital of Wuhan University, Wuhan, China; ^4^Hubei Key Laboratory of Tumor Biological Behaviors, Wuhan, China; ^5^Hubei Cancer Clinical Study Center, Wuhan, China; ^6^The Clinical Medical Research Center of Peritoneal Cancer of Wuhan, Wuhan, China

**Keywords:** bibliometric analysis, NK cells, miscarriage, CiteSpace, VOSviewer

## Abstract

**Background:**

This study aimed to assess the evolution, trends, and research hotspots of publications related to natural killer (NK) cells and miscarriage.

**Methods:**

The literature on NK cells and miscarriage was retrieved from the Web of Science Core Collection. VOSviewer and CiteSpace were used to analyze the publication years, countries, institutions, journals, highly cited authors, categories, and citation bursts of keywords.

**Results:**

A total of 1,275 articles were analyzed. The annual publication outputs showed steady growth, with the majority of publications in 2020 and citations in 2022. The number of publications in this field fluctuated from 1981 to 2023, with a slight downward trend observed. However, the number of citations increased steadily until 2023, followed by a minor decline. The United States contributed the highest number of publications and had the highest h-index. The American Journal of Reproductive Immunology ranked first in terms of number of publications and h-index. Reproductive biology, immunology, and obstetrics and gynecology were the most representative disciplines. Kwak-kim J, Chaouat G, and Croy BA were the top three most productive authors in the field. Keyword burst analysis demonstrated that the immune system and cytotoxicity receptors were current research hotspots.

**Conclusion:**

This is the first bibliometric study to comprehensively summarize trends and advances in the study of NK cells in miscarriage. This information highlights the recent research frontiers and emerging directions and provides a reference for subsequent research in the future.

## 1 Introduction

Spontaneous miscarriage (SM) refers to pregnancies that do not result in a live birth, representing one of the most prevalent adverse pregnancy outcomes, with an incidence rate of 15.3% across all recognized pregnancies (1–3). The primary factors contributing to SM encompass genetic factors, endocrine disorders, anatomical abnormalities, immunological factors, and pre-thrombotic states (4). The role of the immune system in contributing to SM, particularly in cases of recurrent spontaneous abortion (RSA), has garnered significant attention in recent decades (5). Natural killer (NK) cells are a type of lymphocytes with large granular lymphocyte morphology, categorized into CD56^bright^ and CD56^dim^ subtypes based on their intensity, playing a crucial role in the onset and resolution of inflammation, defending against cancer and viral infections (6–12). Peripheral blood NK cells (pNKs) account for approximately 10–15% of the total lymphocytes in peripheral blood, and approximately 90% are CD56^dim^ NK cells (13–15). NK cells presenting in the endometrium are known as uterine NK cells (uNKs), representing 30% of endometrial lymphocytes, while NK cells presenting in the endometrium of pregnant women are known as decidual NK cells (dNKs), which constitute 70% of decidual lymphocytes (16, 17). NK cells are involved in the establishment and maintenance of pregnancy by regulating angiogenesis and trophoblast invasion, remodeling maternal spiral artery via chemokines and cytokines, and protecting the fetus against infectious pathogens (18–20). Dysregulated NK cells are associated with the emergence and development of SM, especially for RSA (21, 22), which were identified by analyzing cell numbers, cell surface receptors, intracellular proteins and mediators, and evaluation of effector functions (23).

Bibliometric analysis is a globally recognized statistical assessment of published articles, providing vital insights and identifying research frontiers for researchers within a specific field (24). Despite the increasing amount of literature on NK cells and miscarriage, there have been limited bibliometric analyses exploring the key research areas and frontiers in the field. In this study, we aimed to provide quantitative information in the global literature on the relationship between NK cells and miscarriage, identifying the trends and potential hotspots by synthesizing and analyzing the relevant information. We briefly discussed NK cell–miscarriage research and predicted potential trends as well as future directions of research and hotspots.

## 2 Materials and methods

### 2.1 Data collection and search strategy

**Figure 1:**
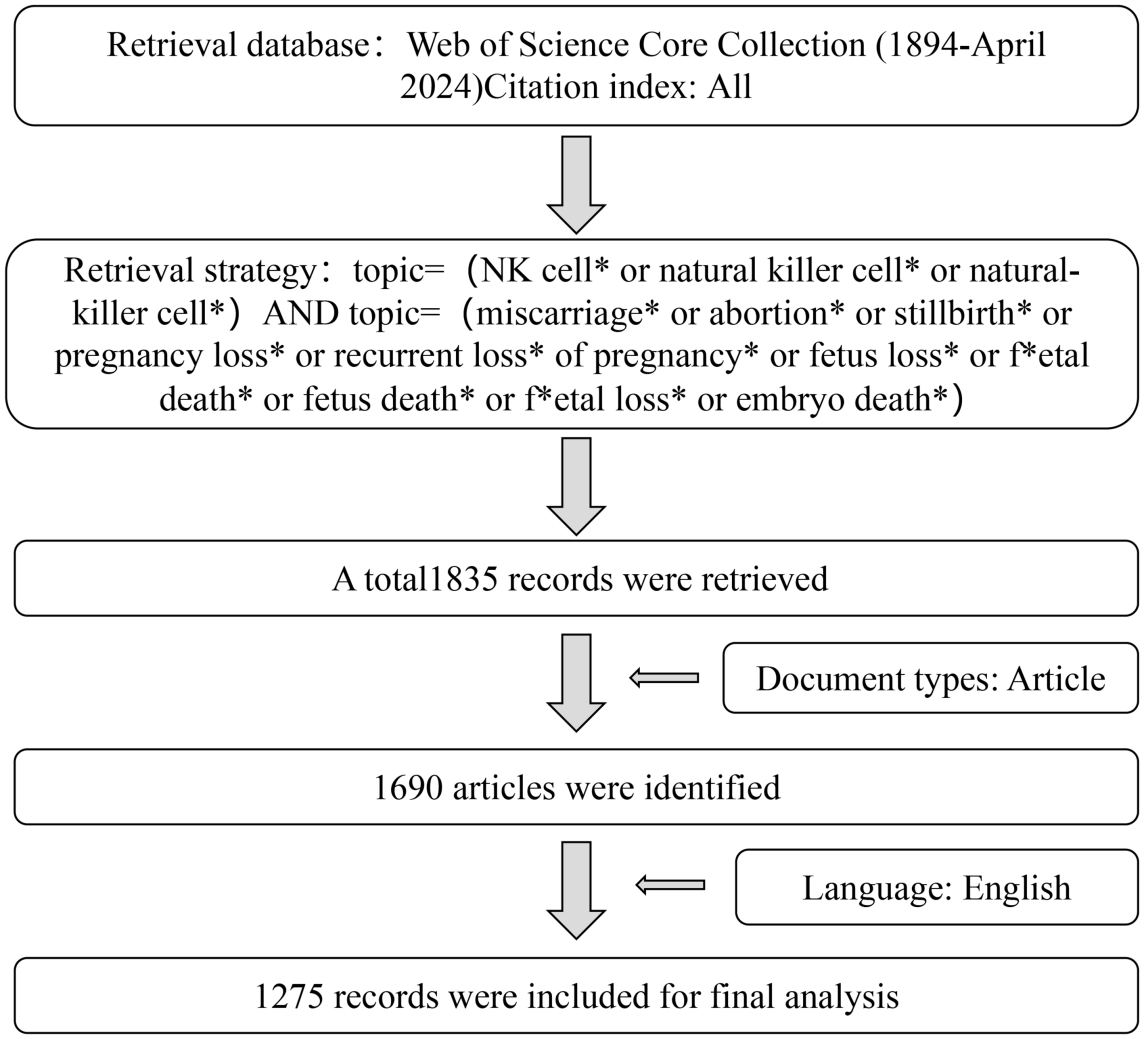
Publications screening flowchart.

All relevant articles published were extracted from the Web of Science Core Collection (WoSCC). The Web of Science (WOS) contains many influential and high-quality journals from all over the world, which are constantly updated and supplemented. It is authoritative in the field of medical research and widely recognized around the world (25). The search strategy was as follows: Topic = (NK cell* or natural killer cell* or natural-killer cell*) and Topic = (miscarriage* or abortion* or stillbirth* or pregnancy loss* or recurrent loss* of pregnancy* or fetus loss* or fetal death* or fetus death* or fetal loss* or embryo death*). To ensure the comprehensiveness of the study data, we conducted a search for all relevant documents included in the WOSCC database, spanning from 1981 to 7 May 2024. A total of 1,835 records were retrieved and extracted for further analysis. To ensure the relevance of the retrieved results to this study, we manually screened the retrieved studies by removing duplicates and retaining only the original literature. A total of 1,690 articles were ultimately screened. Only original articles in English were included, so the remaining 1,275 records were included in the final analysis. We exported the 1,275 records three times, selecting full records and cited references, and saved them as plain text files (Supplementary Table S1). All data were downloaded on 7 May 2024. The flowchart is displayed in Figure 1.

### 2.2 Data extraction and analysis

CiteSpace 6.3.R1 and VOSviewer 1.6.20.0 were used for raw dataset analysis. The “Analyze Search Results” and “Citation Report” functions of the WoS were used to perform basic statistics, including production by year, type of results analyzed, and quality of publications by country, institution, author, and journal. Publication quality in this study mainly referred to the number of publications, the sum of citations, the average number of citations per item (ACI), and the h-index. Bibliometric analysis software and visualization of co-authorship between countries, institutions, authors, and co-occurrence of keywords were conducted with VOSviewer. CiteSpace was used to further analyze the potential links and key themes. The parameters of CiteSpace were set as follows: link retaining factor (LRF = 3), look back years (LBY = 5), years per slice (1), links (strength = cosine; scope = within slices), selection criteria (g-index, *k* = 5), and minimum duration (MD = 1). The contents of the plots produced by the two software programs were appropriately configured and utilized to extract the key information, which was then used to create bar charts and line graphs for enhanced data visualization.

## 3 Results

### 3.1 Temporal distribution map of the literature

**Figure 2:**
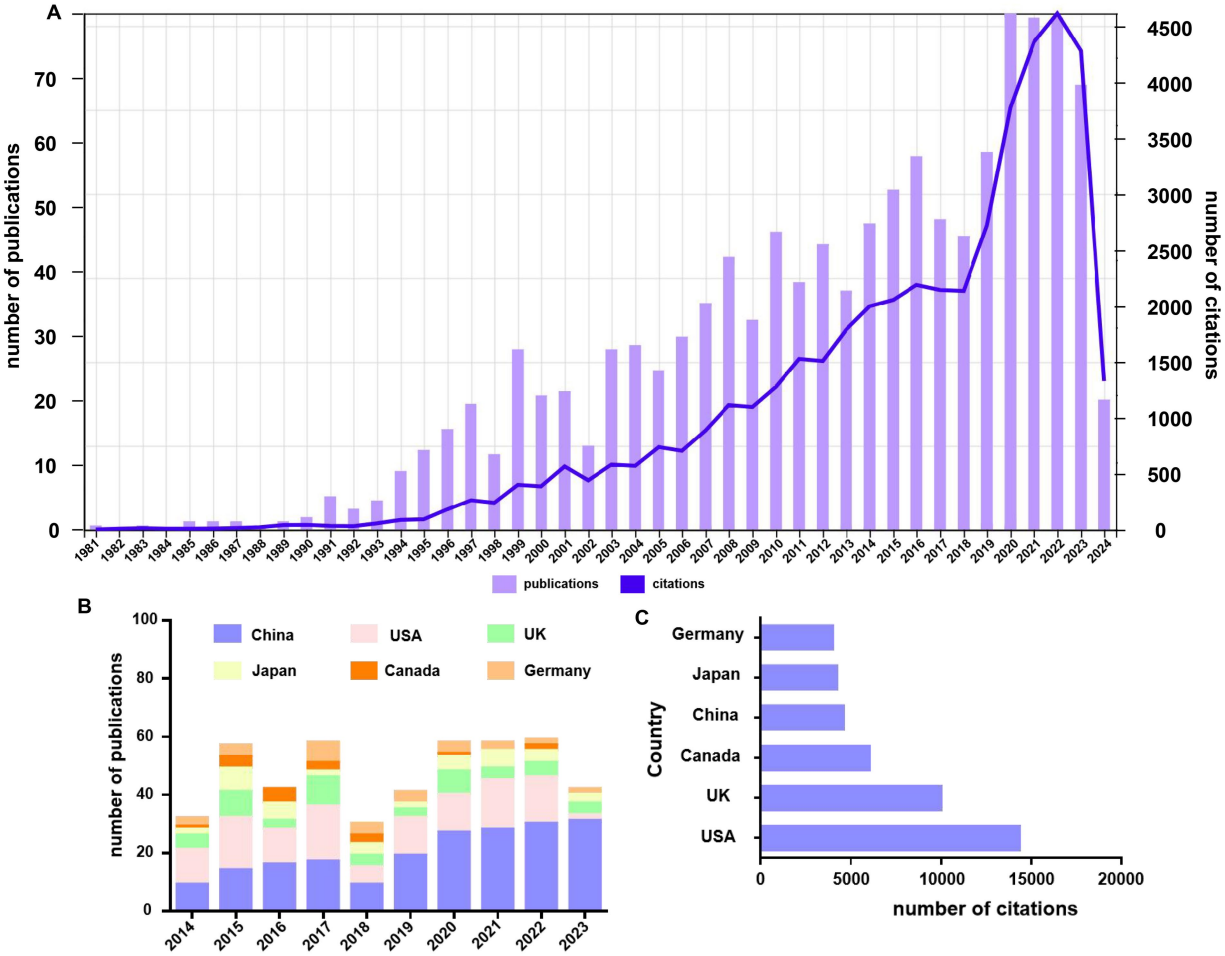
Analysis of the trends and quantity of annual publications. **(A)** Numbers and citations of publications on NK cells and miscarriage from 1981 to April 2024. **(B)** Number of publications and growth trends for the top six countries on NK cells and miscarriage from 2014 to 2023. **(C)** Bar graph of the total number of citations among countries from 1981 to April 2024 for the top six countries with the highest total number of citations. Each bar represents a country, and the length is positively correlated with the total number of citations.

A total of 1,275 pieces of literature on NK cells and miscarriage were retrieved from the WoS. The earliest publication in this field dates back to 1981. As illustrated in Figure 2A, the number of publications exhibits a fluctuating upward trend, peaking at 79 in 2020. Based on the annual publication rate, the research in this field can be divided into three distinct stages. The initial stage (1981–2000) showed a slower growth trend, the second stage (2001–2020) displayed a steady improvement, and the last stage (2021–2023) showed a rapid trend in the field. Between 1981 and 2022, the citation frequency gradually increased, reaching a peak in 2022 (4,637). To analyze which countries were leading the field, we created a bar chart using data from the WOSCC. Figure 2B shows the number of literature published by the top six countries. We found that China had the highest number of publications and that this grew rapidly after 2018. In other countries, the number of publications was erratic. Figure 2C shows the top six countries based on the total number of citations. During this period, the number of citations of literature published in the United States ranked first, with 14,443. The United Kingdom and Canada ranked second and third, with 10,104 and 6,106, respectively. Although China had the second highest number of literature in the world in this field, the number of citations was not as high as the other three countries.

### 3.2 Distribution of countries and institutions

**Figure 3:**
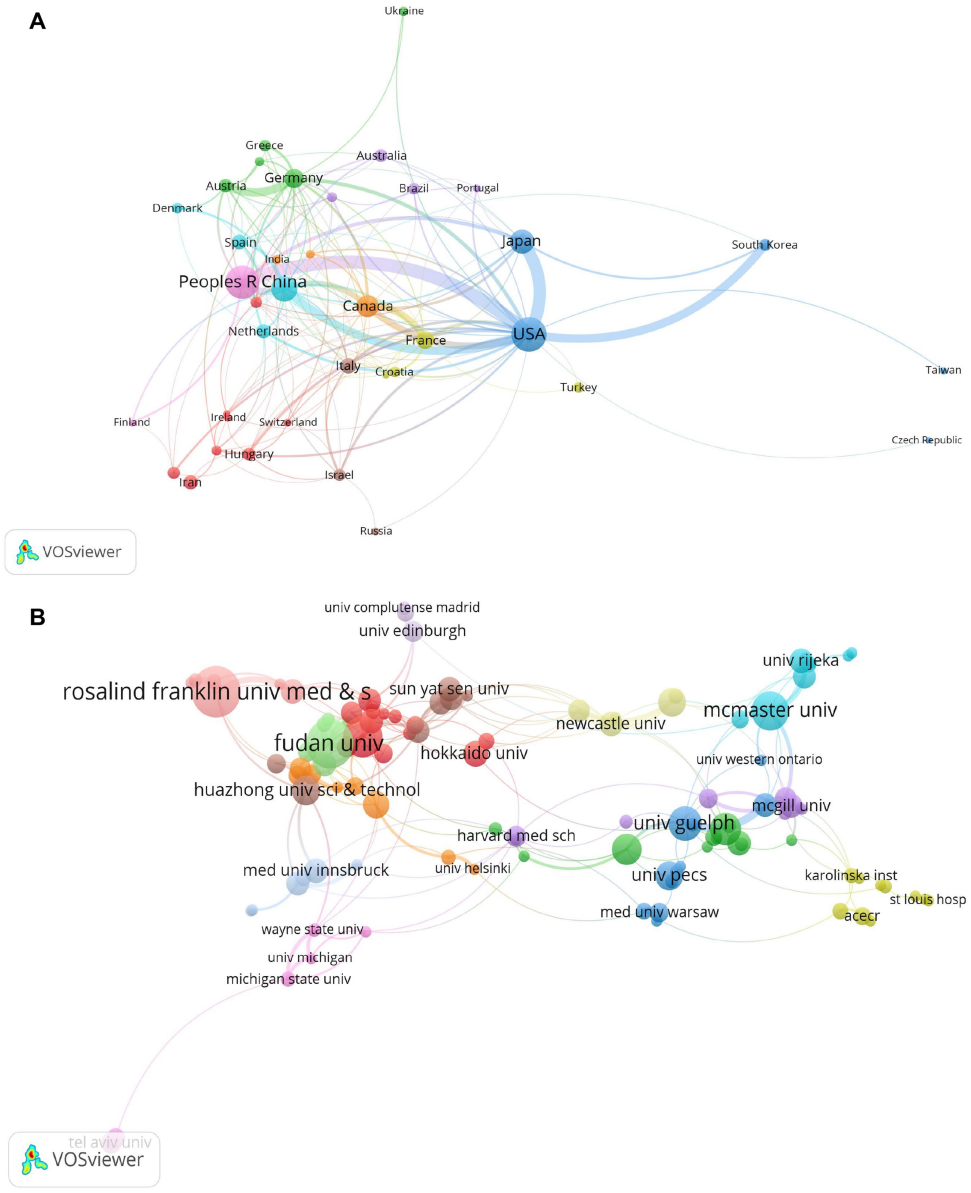
Collaborative network map of countries and institutions. **(A)** Countries with publications of more than 5. **(B)** Institutions with publications of more than 5. The circles represent different countries and institutions, and the size of the circles indicates the number of published articles.

A total of 74 countries published literature in this field. The United States contributed the highest number of publications (290, 22.7%), followed by China (260, 20.4%), the United Kingdom (143, 11.2%), and Japan (125, 9.8%). Regarding the ACI value, the United Kingdom (70.51), Germany (59.57), Canada (58.55), the United States (49.15), and Japan (34.54) ranked in the top five. In terms of the h-index, the United States (63), the United Kingdom (51), and Canada (42) were in the top three countries (Table 1). The top five countries with the highest total link strength were the United States (168), the United Kingdom (89), China (68), Canada (62), and Japan (51) (Table 1). The co-authorship analysis revealed that a total of 34 countries had more than five publications in this field, with the United States having a particularly close collaboration with China, Japan, and South Korea (Figure 3A). To clarify the interinstitutional collaborations, VOSviewer was used to build the visual map of institutions. Table 2 lists the productive institutions with ≥30 publications. Rosalind Franklin University of Medicine Science (39), Institut National de la Santé et de la Recherche Médicale (39), and Apublique Hopitaux Paris Aphp (38) showed the highest number of publications in this field. Harvard University (92.31), Institut National de la Santé et de la Recherche Médicale (54), and Apublique Hopitaux Paris Aphp (51.21) displayed the highest ACI value. VOSviewer revealed that Fudan University (45), Shanghai Jiao Tong University (28), and Sheffield Hallam University (25) had the highest total link strength. In addition, we analyzed the co-authorship of the 122 institutions with more than 5 publishers, and the results showed that Fudan University had more collaboration with other institutions in this field (Figure 3B).

**Table 1:** Top five countries regarding the research on NK cells and miscarriage.

Rank	Country	Quantity	Percentage	ACI	h	Total link strength
1	USA	290	22.70%	49.15	63	168
2	China	260	20.40%	17.93	37	68
3	United Kingdom	143	11.20%	70.51	51	89
4	Japan	125	9.80%	34.54	38	51
5	Canada	101	7.90%	58.55	42	62

ACI, average citations per item; h, h-index.

**Table 2:** Top five organizations with the largest number of publications involved in NK cells and miscarriage.

Rank	Institution	Country	Quantity	STC	ACI	h
1	Rosalind Franklin University of Medicine Science	United States	39	1744	44.72	23
2	Institut National de la Santé et de la Recherche Médicale	France	39	2,106	54	25
3	Apublique Hopitaux Paris Aphp	France	38	1946	51.21	25
4	Fudan University	China	37	923	24.95	16
5	Harvard University	United States	35	3,231	92.31	20

STC, sum of the times cited; ACI, average citations per item; h, h-index.

### 3.3 Analysis of journals and distribution of disciplines

We analyzed 41 journals with more than five pieces of literature on NK cells and miscarriage. American Journal of Reproductive Immunology (215) published the highest number of literature, followed by the Journal of Reproductive Immunology (112) and Human Reproduction (60). Journal of Immunology (107.83) had the highest ACI value, followed by Human Reproduction (70.7) and Biology of Reproduction (50.08). American Journal of Reproductive Immunology (47), Human Reproduction (39), and Journal of Reproductive Immunology (32) displayed the highest h-index (Table 3).

**Table 3:** Top 10 journals for research on NK cells and miscarriage.

Rank	Journal title	Country	Quantity	ACI	2022 IF	Q	h
1	American Journal of Reproductive Immunology	United States	215	32.61	3.6	Q3	47
2	Journal of Reproductive Immunology	Netherlands	112	26.31	3.4	Q3	32
3	Human Reproduction	United Kingdom	60	70.7	6.1	Q1	39
4	Fertility and Sterility	United States	38	36.42	6.7	Q1	23
5	Journal of Immunology	United States	29	107.83	4.4	Q2	26
6	Frontiers in Immunology	Switzerland	27	18.37	7.3	Q1	13
7	Placenta	United Kingdom	27	40.85	3.8	Q2	19
8	Biology of Reproduction	United States	26	50.08	3.6	Q2	19
9	Reproductive Biomedicine Online	United Kingdom	18	31.17	4	Q1	12
10	Plos One	United States	17	30.06	3.7	Q2	13

ACI, average citations per item; IF, impact factors; Q, quartile in the category; h, h-index.

Based on the number of publications, the top three disciplines were Reproductive Biology (588, 46.1%), Immunology (519, 40.7%), and Obstetrics and Gynecology (313, 24.5%). Other disciplines included Cell Biology and Medical Research Experimental and Developmental Biology (Table 4).

**Table 4:** Top 10 categories in the NK cells and miscarriage.

Rank	WOS categories	Quantity	Percentage
1	Reproductive Biology	588	46.1%
2	Immunology	519	40.7%
3	Obstetrics Gynecology	313	24.5%
4	Cell Biology	77	6.0%
5	Medical Research Experimental	63	4.9%
6	Developmental Biology	60	4.7%
7	Multidisciplinary Sciences	56	4.4%
8	Biochemistry Molecular Biology	55	4.3%
9	Genetics Heredity	41	3.2%
10	Endocrinology Metabolism	40	3.1%

### 3.4 Analysis of authors

Kwak-kim J from Rosalind Franklin University of Medicine & Science published the highest number of literature (34), followed by Chaouat G (29) and Croy BA (26). Croy BA from Canada (69.08), Chaouat G (55.48) from France, and Clark DA (50.13) from Canada had the highest ACI value. Of the top 10 authors, 3 were from China, 2 were from the United States, and 2 were from Canada. The remaining three were from France, Japan, and Austria. Chaouat G (21), Kwak-kim J (20), and Croy BA (20) had the highest H-value (Table 5).

**Table 5:** Top 10 authors of studies on NK cells and miscarriage.

Rank	Author	Country	Institute	TP	P	ACI	h
1	Kwak-kim J	United States	Rosalind Franklin University of Medicine & Science	34	2.7	30.56	20
2	Chaouat G	France	Hopital Universitaire Saint-Louis – APHP	29	2.3	55.48	21
3	Croy BA	Canada	Queens University	26	2.0	69.08	20
4	Clark DA	Canada	McMaster University	24	1.9	50.13	16
5	Li DJ	China	Fudan University	23	1.8	36.04	14
6	Gilman-sachs A	United States	Rosalind Franklin University of Medicine & Science	22	1.7	47.18	17
7	Li TC	China	Chinese University of Hong Kong	21	1.6	38.52	14
8	Fukui A	Japan	University of Hyogo	19	1.5	37.37	12
9	Lin Y	China	Jinan University	19	1.5	24.89	14
10	Toth B	Austria	Medical University of Innsbruck	19	1.5	22	13

TP, total publications; P, percentage; ACI, average citations per item; h, h-index.

**Figure 4:**
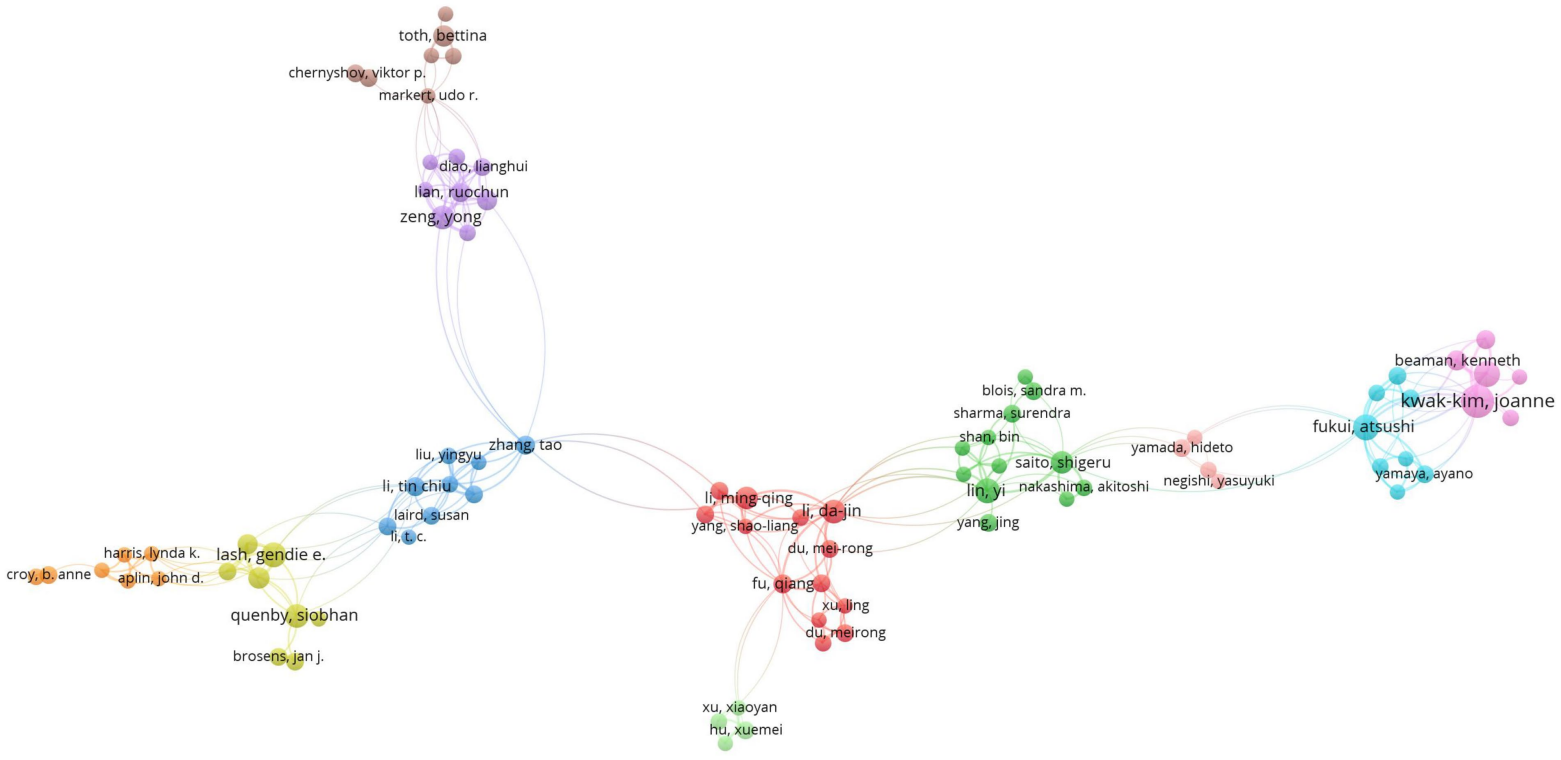
Authors with the highest number of publications (number ≥ 5). Each circle represents an author, and links between the two circles indicate cooperation with each other. Font size is positively correlated with the quantity of published papers.

We analyzed 149 authors who had collaborated with each other with at least 5 articles. VOSviewer showed that Fukui, Atsushi (61), Kwak-kim J (58), and Zeng Yong (48) had the highest total link strength, indicating that these three authors had more partnerships with other authors on NK cells and miscarriage (Figure 4).

### 3.5 Citation analysis

Table 6 lists the top 10 most cited articles. The most cited article was “Single-cell reconstruction of the early human maternal-fetal interface” (26), with 1,185 citations, followed by “Preeclampsia Pathophysiology, Challenges, and Perspectives” (27), with 903 citations.

**Table 6:** Analysis of the top 10 citations in the literature on NK cells and miscarriage.

Rank	Title	Journal	Authors, Year	C	R
1	Single-cell reconstruction of the early maternal-fetal interface in humans	Nature	Vento-Tormo, R et al., 2018 (26)	1,185	70
2	Preeclampsia Pathophysiology, Challenges, and Perspectives	Circulation Research	Rana, S et al., 2019 (27)	903	264
3	Inhibition of allogeneic T cell proliferation by indoleamine 2,3-dioxygenase-expressing dendritic cells: Mediation of suppression by tryptophan metabolites	Journal of Experimental Medicine	Terness, P et al., 2002 (43)	761	44
4	The Transcription Factors T-bet and Eomes Control Key Checkpoints of Natural Killer Cell Maturation	Immunity	Gordon, SM et al., 2012 (44)	548	59
5	GATA2 deficiency: a protean disorder of hematopoiesis, lymphatics, and immunity	Blood	Spinner, MA et al., 2014 (64)	474	58
6	Maternal activating KIRs protect against human reproductive failure mediated by fetal HLA-C2	Journal of Clinical Investigation	Hiby, SE et al., 2010 (65)	368	63
7	Evidence-based guidelines for the investigation and medical treatment of recurrent miscarriage	Human Reproduction	Jauniaux, E et al., 2006 (66)	367	80
8	Evidence for Immune Cell Involvement in Decidual Spiral Arteriole Remodeling in Early Human Pregnancy	American Journal of Pathology	Smith, SD et al., 2009 (67)	346	54
9	Systemic inflammatory priming in normal pregnancy and preeclampsia: The role of circulating syncytiotrophoblast microparticles	Journal of Immunology	Germain, SJ et al., 2007 (68)	346	51
10	Endometrial T, B, and NK cells in patients with recurrent spontaneous abortion-Altered profile and pregnancy outcome	Journal of Immunology	Lachapelle et al., 1996 (30)	323	51

Y, publication year; C, citations; R, references.

**Figure 5:**
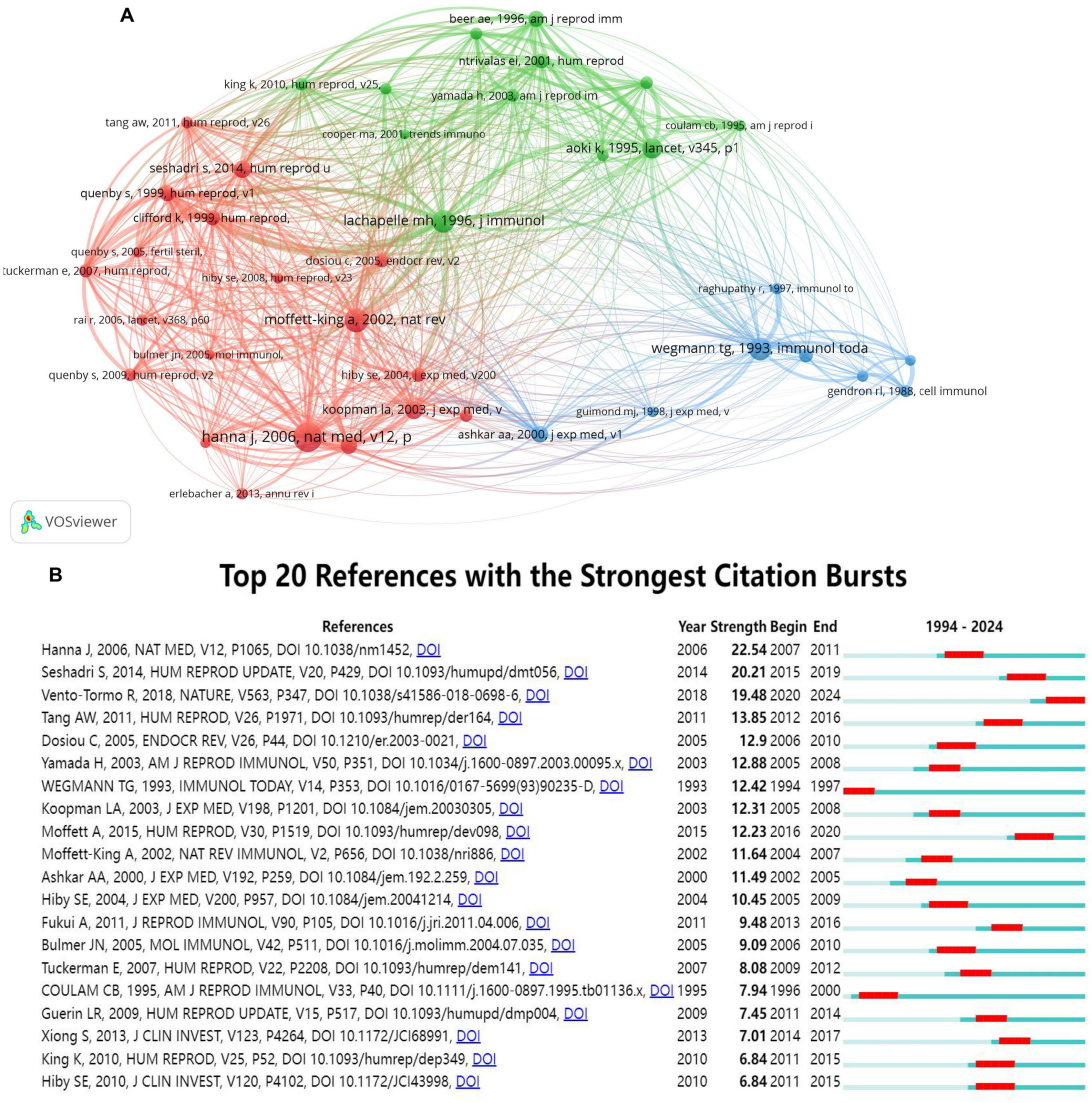
Reference co-citation network analysis of publications on NK cells and miscarriage. **(A)** CiteSpace co-citation map of 39 references in the field. Each node represents a reference, the size of the node is positively correlated with the citation frequency, and the link between two nodes represents two references cited in the same article. **(B)** The top 20 references with the strongest citation bursts for articles in the field. The blue bars indicate the period in which the reference has been published, and the red bars represent bursts of citation frequency.

Figure 5A shows the 39 references with the highest co-citations, which could help the researcher understand the development and evolution dynamics of the field. In the literature co-citation analysis, the higher the co-citation frequency, the closer the academic relationship between these two authors. Lachapelle MH had the closest relationship with other authors, especially with Aoki K and Moffett-king A, based on the total link strength. According to the clustering results, the red clusters were centered on the literature of authors such as Hanna J, the green clusters were centered on articles by authors such as Lachapelle MH, and the blue clusters were centered on articles by authors such as Wegmann TG.

Table 7 lists the top 10 references analyzed by frequency of co-citation. Hanna J *et al*. (180), Moffett-king A (141), Lachapelle MH *et al*. (135), Wegmann TG *et al*. (132), and Aoki K et al. (117) had the largest number of co-citations. In addition, the results showed that the most cited literature was a basic study published in Nature Medicine in 2006. This study demonstrated that dNK cells exerted the ability to regulate placental development including vascular growth and trophoblast invasion (28). This study also discussed the role of chemokines, cytokines, and angiogenic factors of dNK cells at the maternal–fetal interface. The second article was published in 2002, which reviewed the role of uNK cells in reproduction (29). The third article was published in the Journal of Immunology in 1996, which reported decreased CD16^−^CD56^bright^ NK cells and CD8^+^ T cells and increased CD20^+^ B cells in the endometrium of patients with RSA (30).

**Table 7:** Top 10 cited references in the literature on NK cells and miscarriage.

Rank	Cited reference	Citations	Total link strength
1	Hanna J, 2006, NAT MED, V12, P1065, DOI 10.1038/nm1452	180	580
2	Moffett-King A, 2002, NAT REV IMMUNOL, V2, P656, DOI 10.1038/nri886	141	591
3	Lachapelle MH, 1996, J IMMUNOL, V156, P4027	135	663
4	Wegmann TG, 1993, IMMUNOL TODAY, V14, P353, DOI 10.1016/0167-5699(93)90235-d	132	396
5	Aoki K, 1995, LANCET, V345, P1340, DOI 10.1016/s0140-6736(95)92539-2	117	485
6	Koopman LA, 2003, J EXP MED, V198, P1201, DOI 10.1084/jem.20030305	102	411
7	Seshadri S, 2014, HUM REPROD UPDATE, V20, P429, DOI 10.1093/humupd/dmt056	97	403
8	Ashkar AA, 2000, J EXP MED, V192, P259, DOI 10.1084/jem.192.2.259	93	305
9	Bulmer JN, 1991, HUM REPROD, V6, P791, DOI 10.1093/oxfordjournals.humrep.a137430	93	409
10	Quenby S, 1999, HUM REPROD, V14, P2386, DOI 10.1093/humrep/14.9.2386	88	518

The term “references burst” refers to articles that experience a rapid increase in citation frequency, often indicating the emergence or transformation of a research field. In addition, the higher burst strength of the reference shows the greater significance in the field. The top 20 references with the strongest citation bursts were generated using CiteSpace (Figure 5B). The blue line indicated the time frame from 1994 to 2024, and the red line indicated the period over which the burst references were maintained. Among the burst references in recent years, the latest was an article published in Nature in 2018 (26). Among these 20 references, the basic research paper with the highest strength was published in Nature Medicine in 2006, which exhibited the highest burst strength, occurring from 2007 to 2011 (31).

### 3.6 Research hotspots and frontier analysis

Keywords are descriptions of the core content of the articles, and the high frequency of keywords reflects the research hotspots and trends of the main issues in related fields. As shown in Table 8, in addition to NK cells (926), the keywords with the highest frequency of occurrence are miscarriage (756), endometrium (179), T cell (138), cytokines (137), *in vitro* fertilization (137), and lymphocytes (129), indicating that immune mechanisms in miscarriage have gradually attracted researchers’ attention.

**Table 8:** Top 10 keywords in the literature on NK cells and miscarriage.

Rank	Keywords	Occurrences	Total link strength	Rank	Keywords	Occurrences	Total link strength
1	NK cells	926	4420	6	*In vitro* fertilization	137	765
2	Miscarriage	756	3707	7	Lymphocytes	129	717
3	Endometrium	179	1028	8	Infertility	109	682
4	T cell	138	781	9	Decidua	110	660
5	Cytokines	137	780	10	Implantation	106	640

**Figure 6:**
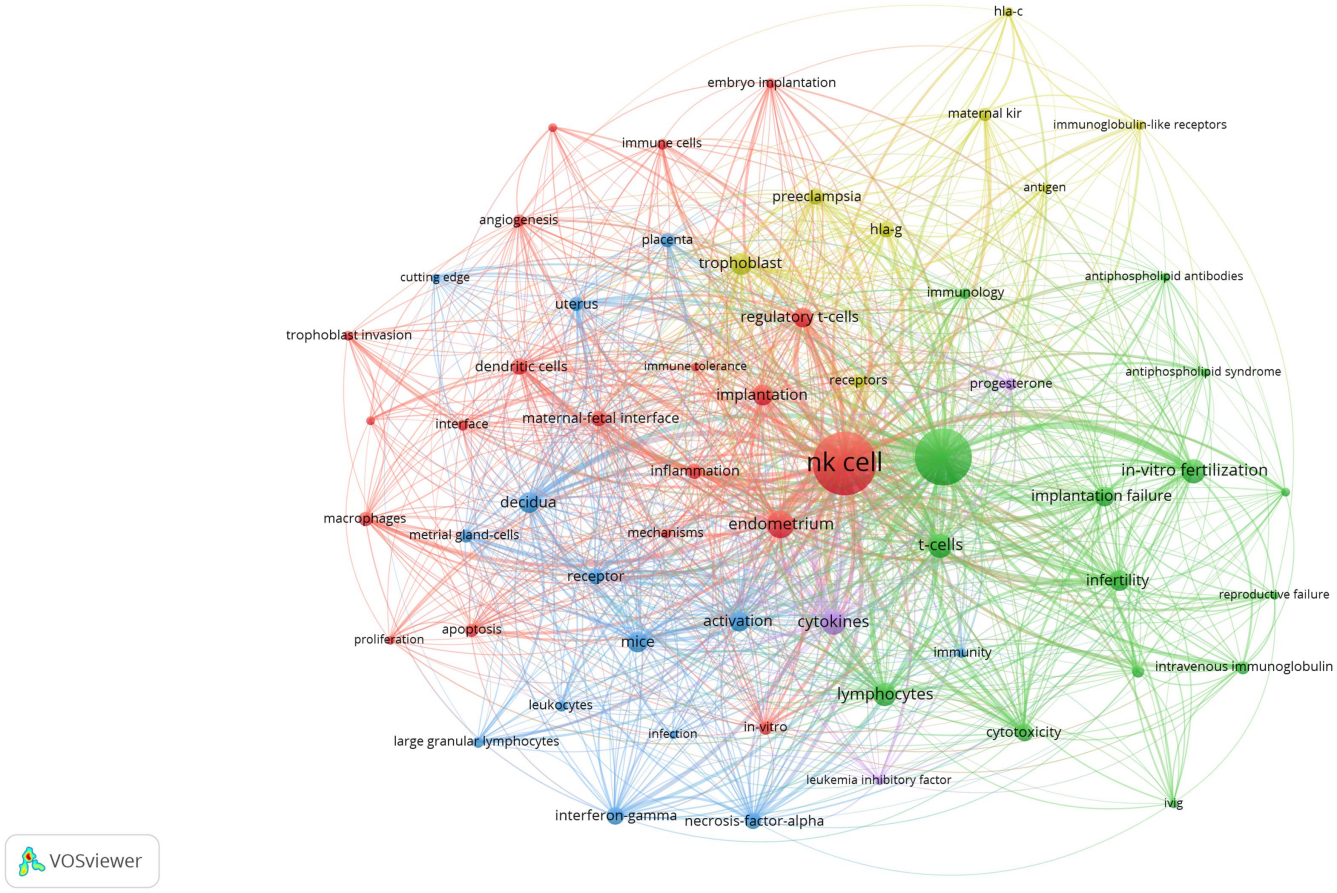
Network visualization of keywords in studies on NK cells and miscarriage. Each node represents a keyword, the size of the node is positively correlated with the number of publications, and the link between two nodes represents two keywords in the same article.

We analyzed a total of 60 keywords occurring more than 20 times after removing the meaningless words and keywords with no significance (Figure 6). The size of the node indicates the number of keyword occurrences, and the thickness of the curve between nodes indicates the frequency of two keyword occurrences at the same time.

The keywords formed five clusters, representing five major research directions in the field. The red clustering groups were dominated by NK cells, endometriosis, regulatory T cells (Tregs), and implantation. The yellow clustering group focused on trophoblast, preeclampsia, and human leukocyte antigen G (HLA-G). The blue clustering group mainly consisted of decidua, mice, and activation. The main research themes of the green cluster were miscarriage, T cells, lymphocytes, and *in vitro* fertilization. The purple clustering focused on cytokines and progesterone.

**Figure 7:**
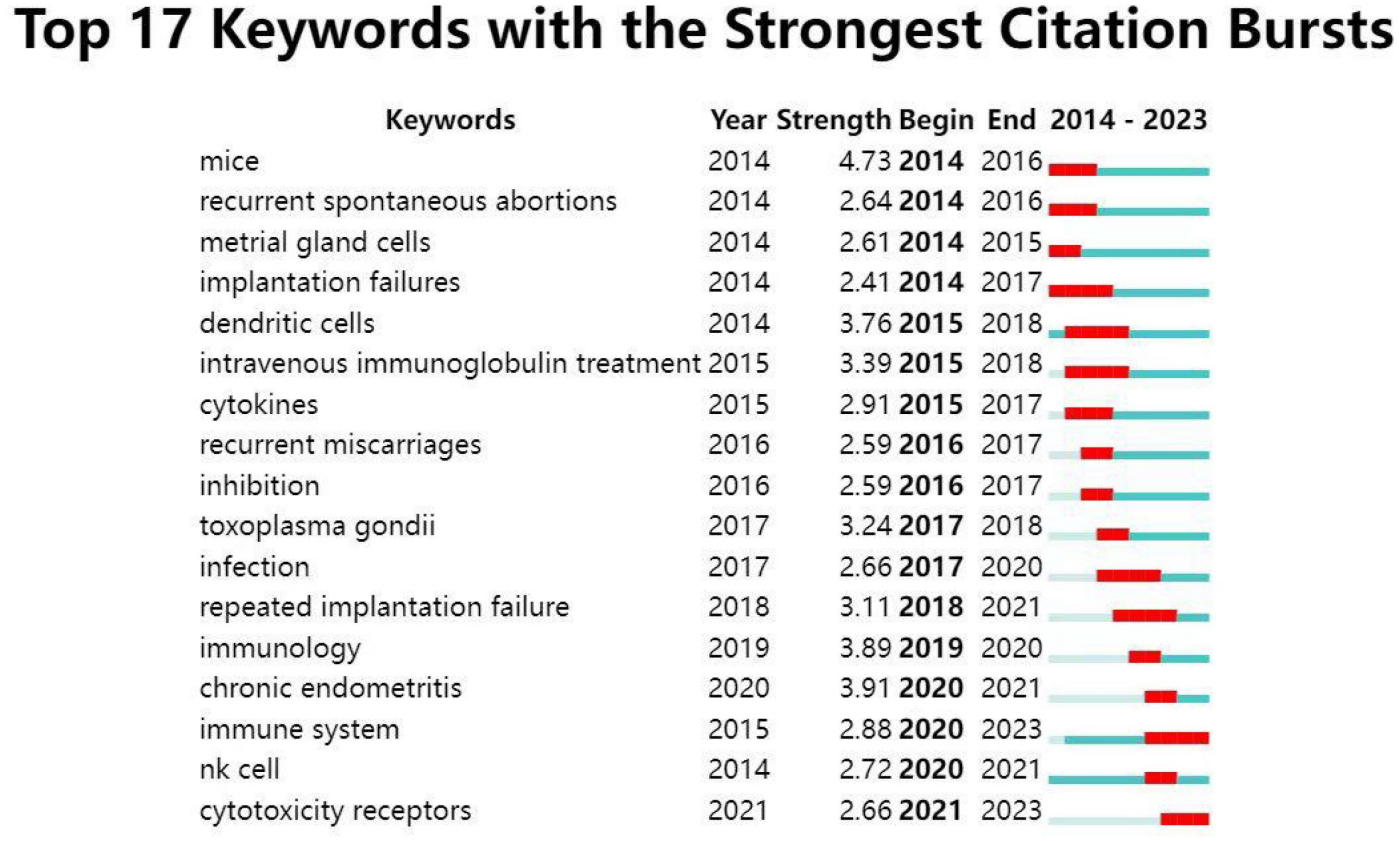
Top 17 keywords with the strongest citation bursts based on CiteSpace. Keywords marked with red bars indicate a sudden increased frequency of use of the keyword during that period, and blue represents a relatively unpopular time period.

Burst patterns of keywords reveal the frontiers and research hotspots of research between NK cells and miscarriage. As shown in Figure 7, the blue line represents the timeline, while the red line indicates the specific ephemeral stage at which the keyword becomes a hotspot of academic research. The light blue indicates nodes that have not yet appeared, and the dark blue shows when the node begins to appear. Of the 17 burst keywords, mice ranked first with the highest burst strength (4.73), followed by chronic endometritis (3.91) and immunology (3.89). Keywords including implantation failure, dendritic cells, intravenous immunoglobulin treatment, infection, and immune system had an outbreak duration of 4 years. The keywords immune system and cytotoxicity receptors are both currently in an outbreak and may be new research hotspots in this field.

## 4 Discussion

This is the first bibliometric and visual analysis of research on NK cells and miscarriage based on CiteSpace and VOSviewer. We retrieved 1,275 publications related to the study of NK cells and miscarriage, published between 1981 and April 2024. The results from the collaboration network analysis show that the United States (Country), Rosalind Franklin University of Medicine Science (institute), American Journal of Reproductive Immunology (journal), and Kwak-kim J (investigator) may be the most influential in the field. Keyword burst detection indicates that the immune system and cytotoxicity receptors are the current research hotspots. Our study will help researchers to identify potential collaborators and collaborating institutions, hotspots, and frontiers in the field.

Research on NK cells and miscarriage has been rising in waves around the world. The number of global literature and cited articles on NK cells and miscarriage has shown an increasing trend, and this number is likely to further increase in the future depending on the trend. On one hand, the etiology of some miscarriages, especially RSA, is unknown. In recent years, more and more studies have begun to focus on the role of immune factors, especially NK cells. On the other hand, the effectiveness of various treatments for disturbed NK cells is controversial and has not yet been elucidated. It has been demonstrated that the administration of prednisolone could reduce uNK cells in RSA women (32), and even the use of prednisolone to prevent future miscarriage has been reported by several studies (33–35). However, the evidence to support prednisolone administration for RSA women to increase the live birth rate is not convincing. In addition, the normal range of uNK and pNK cells has yet to be established, and the correlation between uNK cells and pNK cells remains unclear. Quenby *et al*. used the 75th percentile of 18 fertile controls as the cutoff value of 5% (32), Tuckerman *et al*. used the 90th percentile of 13 fertile controls to define the upper limit value of 12.9% (36), while Chen *et al*. used the 5th and 95th percentiles of 10 fertile controls to define the reference range of 1.2 to 4.5% (37). It has been demonstrated that pNK cells could reflect dNK cells in women with RSA (38, 39), but it is not convincing. Although the studies on NK cells and miscarriage have gradually advanced in recent years, the current research studies are not enough to cover the standardized methods of measuring NK cells, the normal range of NK cells, and the effectiveness of interventions, and research studies in this field have a vast potential for future development with more input and deeper studies.

Over the past 30 years, the United States has been the largest contributor in this field. China has also made significant contributions, with 3 of the top 10 authors, based on the number of published articles, coming from China. In addition, Fudan University in China was one of the institutions with the highest total link strength, contributing numerous articles in this field. The United Kingdom ranked first in terms of ACI, followed by Germany and Canada, indicating the high quality of articles in these countries. Notably, the majority of publishers were from the United States and the United Kingdom except one from Switzerland and one from the Netherlands. Possible reasons for the lack of journals from Asian countries may be non-native English speakers. China needs to create some international journals to strengthen its academic impact. Therefore, these journals, institutes, and countries may continue to make a great contribution in the future.

Kwak-kim J and Chaouat G were the two most prolific authors in this field. In 2001, Kwak-kim J et al. published a study about the status of pNK cells in women with RSA and infertility of unknown etiology. The authors reported that these women had higher proportions of activated pNK cells (40), which have been cited 142 times so far. As the top-cited author, Prof. Croy BA is an internationally well-known expert in the field of reproduction, working primarily at the Queen’s University at Kingston. Chaouat G is a professor at the Department of Medicine, McMaster University. In 1987, Chaouat G and Croy BA collaborated to review the immunological and para-immunological mechanisms in SM (41), along with Clark DA, the third most-cited author. He and colleagues reported the characterization of murine dNK cells and their relevance with pregnancy in 1985 (42), which has been cited 107 times so far. Furthermore, more emphasis should be placed on the contribution of relatively young researchers in the field.

A comprehensive analysis of journals could provide a useful reference for researchers to find literature or appropriate journals for submitting manuscripts quickly. Our results demonstrated that the American Journal of Reproductive Immunology had the highest number of publications, and the Journal of Reproductive Immunology, Human Reproduction, and Fertility and Sterility were the core journals on NK cells and miscarriage. All these journals were focused on the field of reproductive medicine. Therefore, advances and emerging trends in this field are likely to be published in these journals.

The number of citations an article receives can serve as an indicator of its influence and impact. The most highly cited review, titled “Single-cell reconstruction of the early human maternal-fetal interface,” published in Nature, delves into the cellular organization of the decidua and placenta (13). The second highest cited article titled “Preeclampsia Pathophysiology, Challenges, and Perspectives” was published in Circulation Research, which discussed the current evidence of abnormal placentation and the role of placental factors in the pathogenesis of the maternal syndrome of preeclampsia. The authors also described the role of uNK cells in the abnormal placentation of preeclampsia (27). The third article, titled “Inhibition of allogeneic T cell proliferation by indoleamine 2,3-dioxygenase-expressing dendritic cells: Mediation of suppression by tryptophan metabolites,” was published in the Journal of Experimental Medicine, which demonstrated the suppressive mechanisms mediated by dendritic cells (43). An article titled “The Transcription Factors T-bet and Eomes Control Key Checkpoints of Natural Killer Cell Maturation” was published in Immunity, which described two sequential checkpoints of NK cell maturation (44).

The topic of NK cells and miscarriage has developed in a multidisciplinary way, with reproductive biology, immunology, and obstetrics and gynecology at its core, which was reflected in the literature on topics such as the complex and specific immune relationship between the embryo and the mother, the factors affecting reproduction, and the common diseases in gynecology. In addition, cell biology, medical research experimental, developmental biology, multidisciplinary sciences, biochemistry molecular biology, genetics heredity, and endocrinology metabolism should not be ignored. The multidisciplinary approach will help to understand the role of NK cells in miscarriage to guide the prevention and treatment. Moreover, the involvement of basic disciplines will gain insight into an understanding of the mechanism.

The emergence or transformation of a research field can be identified by reference bursts. The latest burst reference was an article published in Nature in 2018, which profiled the cellular composition of decidua and placenta (26). The second reference was a review published in Human Reproduction in 2015, and the burst occurred from 2016 to the end of 2020 (45). This study reviewed the function, measurement, and range of uNK cells. The third reference was a meta-analysis published in Human Reproduction Update in 2014, and the burst occurred from 2015 to the end of 2019 (31). This study analyzed the number/percentage of uNK and pNK cells in infertile versus fertile women, as well as RSA versus controls. Among these 20 references, the basic research paper with the highest strength was published in Nature Medicine in 2006, and this burst occurred from 2007 to 2011.

The top 17 keywords with the strongest bursts in the field over the past 30 years were analyzed. The strongest keyword was “mice,” indicating the important role of the mice model in NK cells and miscarriage. It is obvious that numerous experiments in mice cannot be conducted in humans, which can offer an insight into the pathogenesis of miscarriage, NK cell biology, and even therapeutic effects. Several mice models have been used to investigate the mechanisms and treatment of miscarriage, such as the CBA/J × DBA/2 model and lipopolysaccharide-induced model (46). The second keyword was “chronic endometritis,” an issue related to miscarriage that scholars have paid more attention to in recent years. In women with chronic endometritis, alterations in the cellular and biochemical processes, as well as uterine contractility may cause disordered endometrial receptivity, leading to miscarriage (47, 48). In addition, the research on immune system and cytotoxic receptors is a hot spot that deserves our attention in this field. Immunological abnormalities are often considered a potential underlying cause of RSA, and numerous studies have explored the relationship between various immune cells and RSA (49). The immune cells, i.e., NK cells, have also become a hot research topic. It has been suggested that successful pregnancy is closely related to the immunoreactivity of cytotoxic NK cells. Among them, the effect of activation or inhibition of killer immunoglobulin-like receptors (KIRs, receptors of NKs that comprise both inhibiting and activating receptors) on NK cells in miscarriage has been more extensively studied (38). The majority of pNK cells are CD56^dim^, which express high levels of CD16 and KIRs, are highly cytotoxic (50). CD56^bright^CD16^dim^ dNK cells are less cytotoxic and also express a range of receptors, including the KIR family and the CD94/NKG2 family, a possible mechanism by which the dNK cells interact with the trophoblast (51). Ligands for these receptors include HLA-C, HLA-E, and HLA-G, which are expressed on trophoblast. Of these, only HLA-C is highly polymorphic and can interact with KIRs expressed on the surface of NK cells (52, 53). The role of the receptor–ligand interactions is to activate NK cells and thereby stimulate the secretion of vascular-modifying cytokines, and/or to inhibit NK cells (by inhibiting the receptor) and thereby protect the trophoblast from NK-mediated lysis (54). Studies have reported that activating KIR genes may be a risk factor for RSA in Chinese women (55); however, the precise role of KIRs in RSA is still unclear, and there is a lack of consensus. Some studies reported activating KIRs, while others reported inhibiting KIR receptors in RSA (54, 56). Since there is considerable genetic variation in the KIR inventory between individuals, a specific KIR pool may predispose an individual to RSA (54). Hence, further studies are required to establish a clear role of KIRs, and NK abnormalities in RSA. In addition to RSA, KIRs also play important roles in other pregnancy complications. Improper binding of KIRs to HLA-C may lead to insufficient activation of dNK cells, resulting in preeclampsia (57). Patients with preeclampsia had more inhibitory KIRs (58) and less activating KIRs (59). In addition, the interaction of the activating KIRs with HLA-C2 had a positive effect on birth weight, while the interaction of the inhibiting KIRs with HLA-C2 displayed a negative effect (60). Continuous efforts to elucidate the specific mechanisms of the role of NK cells and even the immune system in miscarriage would be beneficial in solving the challenge of miscarriage. Future research hotspots may focus on immunological studies, such as the use of immunotherapy and combination therapies to obtain higher therapeutic effects in the field.

Although many studies have confirmed the role of NK cells in physiological pregnancy and miscarriage, there is currently no consensus or guidelines to recommend NK cell testing or treatment for patients with miscarriage, including RSA. The American Society for Reproductive Medicine (61) did not recommend circulating CD16 NK (62) cell testing, the Royal College of Obstetricians and Gynaecologists (62) showed that pNK/uNK cell tests should not be offered routinely in the investigation of RSA, and the European Society of Human Reproduction and Embryology (63) indicated that there was no insufficient evidence to recommend NK cell testing. This may be partly attributed to the fact that the detection methods, reference values, and treatment of NK cells are not coincident at present. Therefore, the role of NK cells in miscarriage needs to be confirmed by more large-scale, multi-center and high-quality clinical studies, so as to provide more high-level evidence support for its clinical use.

However, there are some limitations. First, we only selected the WoSCC from the WoS database to ensure the credibility of the literature. Second, we only analyzed articles in the classical sense, not including reviews. In addition, only publications in English were included in the study, which would undoubtedly miss some excellent findings written in other languages from non-English speaking countries. Some of the articles published recently were not heavily cited, resulting in the possible omission of the hotspot analyses.

## 5 Conclusion

This is the first bibliometric analysis exploring the research on NK cells and miscarriage. With the help of visual analysis software, we analyzed the publication trends, identified the countries, authors, and publications that contributed to the field, and predicted the future research hotspots in the field. Over the last 20 years, the number of publications has increased rapidly, demonstrating that there is a growing interest in the field. The current research hotspot is focused on the immune system and cytotoxicity receptors, suggesting that research studies on the immune system especially cytotoxicity receptors may be the promising option for miscarriage. The study defines the overall prospects in this field and provides important information, including potential collaborators and partner organizations, as well as hotspots and emerging frontiers. It provides useful references for future clinical trials and basic science research.

## Data Availability

The original contributions presented in the study are included in the article/Supplementary material, further inquiries can be directed to the corresponding authors.
