# Supplementary material for: Global research on NK cells in miscarriage: a bibliometric study
Source: Front Med (Lausanne). 2025 Feb 17;12:1513213. doi: 10.3389/fmed.2025.1513213 (PMC11872723; doi:10.3389/fmed.2025.1513213)
Supplement: Supplementary file 1 [file Supplementary_file_1.docx]

| **Table S1** | | | | | | | | | | | | | | | |
| --- | --- | --- | --- | --- | --- | --- | --- | --- | --- | --- | --- | --- | --- | --- | --- |
| **Rank** | **WOS** | **Rank** | **WOS** | **Rank** | **WOS** | **Rank** | **WOS** | **Rank** | **WOS** | **Rank** | **WOS** | **Rank** | **WOS** | **Rank** | **WOS** |
| 1 | 000402534200024 | 161 | 000804969900004 | 321 | 000548503300001 | 481 | 000084708600035 | 641 | 000306308200009 | 801 | 000168660300020 | 961 | 000342744500006 | 1121 | 000254300500010 |
| 2 | 000987380500007 | 162 | 000387855300002 | 322 | 000313909100010 | 482 | 000406634500091 | 642 | 000346271000002 | 802 | 000976398900008 | 962 | 000399472200013 | 1122 | 000282789200003 |
| 3 | 000349411400001 | 163 | 001073816400001 | 323 | 000486426600008 | 483 | 000468781400013 | 643 | 000826723500002 | 803 | 000178266200023 | 963 | 000263755300012 | 1123 | A1995QG31700006 |
| 4 | 000353710000012 | 164 | 000443666100038 | 324 | 000422712600012 | 484 | 000512154100001 | 644 | 000186801700003 | 804 | 000507342400008 | 964 | 000221288300001 | 1124 | 000266925000014 |
| 5 | 000849133700004 | 165 | 000520291300001 | 325 | 000332468000008 | 485 | 000084158500002 | 645 | 000394802500011 | 805 | A1997XK38200002 | 965 | 000435725300001 | 1125 | A1994PJ42800043 |
| 6 | 000625688300001 | 166 | 000405934700010 | 326 | 000386944600017 | 486 | 000416159300013 | 646 | 000341823000008 | 806 | 000831385100001 | 966 | 000864321500011 | 1126 | 000433891800001 |
| 7 | 000464986300004 | 167 | 000416950700014 | 327 | 000080864200003 | 487 | 000183434000003 | 647 | 000570586700001 | 807 | A1994NP91000002 | 967 | 000184634700024 | 1127 | 000750869500001 |
| 8 | 000407915600122 | 168 | 000277608700021 | 328 | 000414326900002 | 488 | 000243789000002 | 648 | 001192210300001 | 808 | 000909864100001 | 968 | 000240433000034 | 1128 | 000399565100003 |
| 9 | 000417125500002 | 169 | 000958425900003 | 329 | 000293324100016 | 489 | 000078065500014 | 649 | 000387734600023 | 809 | 000236461500007 | 969 | A1997WU32600028 | 1129 | 000719420900004 |
| 10 | 000459314500153 | 170 | 000347264300017 | 330 | 000252498700022 | 490 | 000354390000008 | 650 | 000566483200001 | 810 | 000708239900008 | 970 | 000453872300001 | 1130 | 000351919100012 |
| 11 | 000460878100023 | 171 | 000381324400006 | 331 | 000571259400001 | 491 | 000266256700052 | 651 | 000234134300004 | 811 | 000344870400004 | 971 | 000340702100018 | 1131 | 000910947100001 |
| 12 | 001029742100001 | 172 | 000497128300013 | 332 | 000271519800005 | 492 | A1997XW17400008 | 652 | 000287264400007 | 812 | 000235711300008 | 972 | 000387448300138 | 1132 | 000651034900033 |
| 13 | 000383817500012 | 173 | 000278395800006 | 333 | 000241728400005 | 493 | 001208921700008 | 653 | 000334160900142 | 813 | 000246449400005 | 973 | 000357153500001 | 1133 | 000380912800007 |
| 14 | 000231900800001 | 174 | 000232802300005 | 334 | 000083524500013 | 494 | 000250201300011 | 654 | 000273024800018 | 814 | 000181513700007 | 974 | 000923573300001 | 1134 | 000261903400012 |
| 15 | 000580632100031 | 175 | 000373459100003 | 335 | 000275029400005 | 495 | 000309090200011 | 655 | 000271045300002 | 815 | 000249999400002 | 975 | 000890351400001 | 1135 | 000334672400016 |
| 16 | 000348352400006 | 176 | 000435179500008 | 336 | 000890883400002 | 496 | 000307754700007 | 656 | 000266683200006 | 816 | 000330842800005 | 976 | 000089181600013 | 1136 | 000248345400030 |
| 17 | 000072005200011 | 177 | 000326925800010 | 337 | 000332468000007 | 497 | 000258625800005 | 657 | 000226393900010 | 817 | 000366401000024 | 977 | 000895928900003 | 1137 | 000347711000015 |
| 18 | 000834354500002 | 178 | 000580106000001 | 338 | A1997XY74300009 | 498 | 000322939400002 | 658 | 000236902300008 | 818 | 000580630300005 | 978 | 000627873300009 | 1138 | 000231024500005 |
| 19 | 000611876900002 | 179 | 000401137200034 | 339 | 000334114800036 | 499 | A1997YK84500005 | 659 | 000266801400060 | 819 | 000263670600009 | 979 | 000253431400003 | 1139 | 000324316600008 |
| 20 | 000319559100019 | 180 | 000235381600011 | 340 | 000674223800005 | 500 | 000327533000032 | 660 | 000252416400011 | 820 | 000309584900012 | 980 | 000185826100009 | 1140 | 001074387300001 |
| 21 | 000334141300002 | 181 | 000081967300007 | 341 | 000371587400085 | 501 | 000568884100001 | 661 | 000497366400003 | 821 | 000447569700006 | 981 | 000220486800041 | 1141 | 000086677700013 |
| 22 | 000285057600006 | 182 | 000075514600006 | 342 | 000670008400001 | 502 | 000785276200001 | 662 | 000258625800012 | 822 | 000831253500001 | 982 | 000239487500006 | 1142 | 000081759500004 |
| 23 | 000348425400007 | 183 | 000071940900004 | 343 | 001103658100001 | 503 | 000565081900001 | 663 | 000273721200011 | 823 | 000382809700009 | 983 | 000270610900005 | 1143 | 001101912200001 |
| 24 | 000264045000023 | 184 | 000240965600004 | 344 | 000476723100006 | 504 | 000465582800004 | 664 | 000417981200021 | 824 | 000459838500001 | 984 | A1992JM71300016 | 1144 | 000636197100001 |
| 25 | A1991GR22400002 | 185 | 000080901500001 | 345 | A1995RF54000005 | 505 | 000471169800001 | 665 | 000343952000031 | 825 | 000272864100009 | 985 | 000365370500016 | 1145 | A1996VV46300011 |
| 26 | 000086864800025 | 186 | 000567529200011 | 346 | A1988N154400004 | 506 | 001161624100001 | 666 | 000660101300001 | 826 | 000180263700008 | 986 | 000363178200001 | 1146 | 000250815000001 |
| 27 | 000373035100009 | 187 | 000079086700002 | 347 | A1992JB02800011 | 507 | 000752610300012 | 667 | 000770234200002 | 827 | 000338628400009 | 987 | 000474731800008 | 1147 | 000819371000001 |
| 28 | 000603982100001 | 188 | 000365370500003 | 348 | 000785692000001 | 508 | 000279610700005 | 668 | 000567620600001 | 828 | 000253977200003 | 988 | 000318072700012 | 1148 | A1997WM43500063 |
| 29 | 001153350800007 | 189 | 000226513500002 | 349 | 000246054400061 | 509 | 000734918800002 | 669 | 000456635900003 | 829 | 000273569800021 | 989 | 000423711100013 | 1149 | 000075164400005 |
| 30 | 000187565700014 | 190 | 000071915200003 | 350 | 000328947300009 | 510 | 000367331200016 | 670 | 000416226400003 | 830 | 001091417400001 | 990 | A1995QQ15000013 | 1150 | 000263855700006 |
| 31 | 000186120500007 | 191 | 000175326400004 | 351 | 000342505800003 | 511 | 000726312200001 | 671 | 000529344700007 | 831 | 000088565500021 | 991 | 000484820200001 | 1151 | 000238003200011 |
| 32 | 000913030400001 | 192 | A1994MZ18500013 | 352 | 000624565500012 | 512 | 000334295600007 | 672 | 001118966200001 | 832 | 000185826100004 | 992 | A1996TQ19500004 | 1152 | 000243299600014 |
| 33 | 001102938100001 | 193 | 000236645200001 | 353 | 000613289300001 | 513 | 000819118600001 | 673 | 001144391800001 | 833 | 000301868000014 | 993 | 000372001300009 | 1153 | 000222720900005 |
| 34 | 000306918700054 | 194 | 000168929700001 | 354 | 000171442600019 | 514 | 000338932300038 | 674 | 000531532700010 | 834 | 001014710700001 | 994 | 000265551500039 | 1154 | 000534443900001 |
| 35 | 001167650600011 | 195 | 000364913300001 | 355 | 000401511100008 | 515 | 000285205400009 | 675 | 000368140100056 | 835 | 000273449400010 | 995 | 000487092600002 | 1155 | 000468377800011 |
| 36 | 000230168000007 | 196 | 000166926300010 | 356 | 000358015200004 | 516 | 000228926700001 | 676 | 000394475600006 | 836 | 000298788300005 | 996 | 000729740800003 | 1156 | 000302273100007 |
| 37 | 000170129400001 | 197 | 000745655600004 | 357 | 000079086700012 | 517 | 000378842700004 | 677 | 000476804200171 | 837 | 000405934700001 | 997 | A1996UK62100003 | 1157 | 000458365600044 |
| 38 | 000088461000001 | 198 | 000253682000003 | 358 | 000418594400019 | 518 | 000085512100004 | 678 | A1993MC96100015 | 838 | A1991FH28300013 | 998 | 001202080100001 | 1158 | 000404887900009 |
| 39 | A1995RV64000010 | 199 | 000083600200011 | 359 | 000340530100003 | 519 | A1996WE61500005 | 679 | 000810957300001 | 839 | 000296214200017 | 999 | 000340899200006 | 1159 | 000294873600011 |
| 40 | 000169812000036 | 200 | 000358815000004 | 360 | 000178187400008 | 520 | 000078288400006 | 680 | 000382337900093 | 840 | 000298281100006 | 1000 | 000177107900008 | 1160 | 000262451700003 |
| 41 | 000375461800014 | 201 | 000294039900001 | 361 | 000341826700029 | 521 | 000080247900008 | 681 | 000632487800003 | 841 | 000184683300007 | 1001 | 000335834600006 | 1161 | 000296031500005 |
| 42 | 000367552200015 | 202 | 000369169300025 | 362 | 001068347200001 | 522 | 000272929600010 | 682 | 000948189400001 | 842 | 001031577500001 | 1002 | 000248720900022 | 1162 | 000357906600003 |
| 43 | 000081649100024 | 203 | 000640791800001 | 363 | 000469759100030 | 523 | A1994QH80500005 | 683 | 001134892500001 | 843 | 001163729100085 | 1003 | 000326132300014 | 1163 | 000327139300005 |
| 44 | 000577677000001 | 204 | 000458943700001 | 364 | 000309831500071 | 524 | 000258625800006 | 684 | 001051598600006 | 844 | 000250388000054 | 1004 | 000327180600019 | 1164 | 000224703500035 |
| 45 | 001015680700001 | 205 | 000303373300010 | 365 | 000231369200021 | 525 | 000311644700015 | 685 | 000086913800010 | 845 | 000331976000004 | 1005 | 000165622400012 | 1165 | 000360982400001 |
| 46 | 000463371200010 | 206 | 000221556700009 | 366 | 000401767700005 | 526 | 000168654500023 | 686 | 000872145600001 | 846 | 000360994900017 | 1006 | 000085445800018 | 1166 | 000229332200004 |
| 47 | 000455960300003 | 207 | 000413750800001 | 367 | 000299766000010 | 527 | A1995TJ14100008 | 687 | 000495843100001 | 847 | 001029596000001 | 1007 | 000233410600010 | 1167 | 000345948700053 |
| 48 | 000598157300001 | 208 | 000546270600001 | 368 | 000280117800002 | 528 | 000244771600008 | 688 | 000583436500001 | 848 | 000441171600010 | 1008 | 000611312000001 | 1168 | 000920866100001 |
| 49 | 000482110000042 | 209 | A1995TC86700003 | 369 | 000089463100014 | 529 | 000664974500010 | 689 | 000459710100060 | 849 | 000387115900001 | 1009 | 000260384100004 | 1169 | 000387855300005 |
| 50 | 000473295900018 | 210 | A1995QQ15000006 | 370 | 000353856700006 | 530 | 000721573200001 | 690 | 000295496100012 | 850 | 000357451500018 | 1010 | 000424281600045 | 1170 | 000498326400005 |
| 51 | A1997XG42300011 | 211 | 000178740600006 | 371 | 000320846500012 | 531 | 000676689000001 | 691 | 000328079400012 | 851 | 000250622900013 | 1011 | 000966149400001 | 1171 | 000732529800001 |
| 52 | A1996TQ19500007 | 212 | 000180046800002 | 372 | 000234154000008 | 532 | 000445917400008 | 692 | 000326596100007 | 852 | 000302669900005 | 1012 | 000226513500006 | 1172 | 001033117700001 |
| 53 | A1994PT07100029 | 213 | 000185826100010 | 373 | 000379256100011 | 533 | 000074269700047 | 693 | 000511422800001 | 853 | 000255315700019 | 1013 | 000186801700008 | 1173 | 000322377400041 |
| 54 | 000459768100001 | 214 | 000359674200017 | 374 | 000306760000029 | 534 | 000415005900006 | 694 | 000222118900006 | 854 | 000235063700008 | 1014 | 000230725100042 | 1174 | 000231369200014 |
| 55 | 000472206500001 | 215 | 000274493900004 | 375 | 000360592900024 | 535 | 000474267000003 | 695 | 000186441800007 | 855 | 000256720000007 | 1015 | 000829796900002 | 1175 | 000926306500002 |
| 56 | 000373459100002 | 216 | 000571442200007 | 376 | 000416879100029 | 536 | 000635216100101 | 696 | 001023060100066 | 856 | 000288025000006 | 1016 | 000368529100064 | 1176 | 000405782100016 |
| 57 | 000320322400052 | 217 | 000391090800053 | 377 | A1997WB27000020 | 537 | 000281252000049 | 697 | 000766922200002 | 857 | 000423781200004 | 1017 | 000318612400046 | 1177 | 000352138400011 |
| 58 | 000289451800011 | 218 | 000187565700035 | 378 | 000071729000008 | 538 | 000348430100008 | 698 | 001106402800001 | 858 | 000390722900008 | 1018 | 000090002900005 | 1178 | 000314662400028 |
| 59 | 000257898100059 | 219 | 000245690800002 | 379 | A1997XM90500003 | 539 | 000086507700058 | 699 | 000515443300020 | 859 | 000632651400004 | 1019 | 000701749600009 | 1179 | 000859708500001 |
| 60 | 000880858100001 | 220 | 000386773800004 | 380 | 000380033700005 | 540 | 000325819400079 | 700 | 000525834200001 | 860 | 000322342200008 | 1020 | 000337625300004 | 1180 | 000073738000005 |
| 61 | 000313317300034 | 221 | A1997YJ28100004 | 381 | 000445327800008 | 541 | 000236461500003 | 701 | 000947055800001 | 861 | 000291570000003 | 1021 | 000253329000007 | 1181 | 000352355400022 |
| 62 | 000652369500012 | 222 | A1997XG42300010 | 382 | 000930144900001 | 542 | 000647789600059 | 702 | 000255006200009 | 862 | 000261680600023 | 1022 | A1995TG06500003 | 1182 | 000355364600008 |
| 63 | 000232087600013 | 223 | A1993LE04300001 | 383 | 000320188700002 | 543 | 000252006400015 | 703 | 000697380400007 | 863 | 000257148500006 | 1023 | A1996UJ22500009 | 1183 | 001027980000001 |
| 64 | 000183674400008 | 224 | 000502337600013 | 384 | 000081104000006 | 544 | 000534300500006 | 704 | 000293324100015 | 864 | 000175197000007 | 1024 | A1991FN35200022 | 1184 | 000170129400003 |
| 65 | A1996VK56500013 | 225 | 000525676400001 | 385 | 000428209800011 | 545 | 000383040200019 | 705 | A1994PY18600004 | 865 | A1995RM68500028 | 1025 | A1997XB13700012 | 1185 | 000181546200029 |
| 66 | 000265129700011 | 226 | 001023068700001 | 386 | 000604504300001 | 546 | 000357160300010 | 706 | 000241404000008 | 866 | 000080367800002 | 1026 | A1997XW17400007 | 1186 | 000185826100012 |
| 67 | A1992JB90700003 | 227 | 000608236900002 | 387 | 000282137300013 | 547 | A1996TP36600030 | 707 | 000733630900007 | 867 | 000186767200040 | 1027 | 000255775700161 | 1187 | 000220177900009 |
| 68 | 000283255400001 | 228 | 000174843900002 | 388 | 000404296800001 | 548 | 000316434100009 | 708 | 000386706400012 | 868 | 000173682200012 | 1028 | 000297920700002 | 1188 | 000230786200019 |
| 69 | 001101148300001 | 229 | 000501250400001 | 389 | 001036824500017 | 549 | 000516734200001 | 709 | 000579203200001 | 869 | 000249096600027 | 1029 | 000083449300003 | 1189 | 000466389900002 |
| 70 | 000262696500011 | 230 | 000220255600021 | 390 | 000328526200011 | 550 | 000298306700018 | 710 | 000925739600001 | 870 | 000168454400010 | 1030 | A1997YG67300006 | 1190 | 000823118500001 |
| 71 | 000438337700001 | 231 | 000390824200019 | 391 | 000984274100022 | 551 | 000827902500008 | 711 | 000519448900001 | 871 | 000230762300018 | 1031 | 000286381200023 | 1191 | 000080624500006 |
| 72 | 000275509200004 | 232 | 000183190700060 | 392 | 000850237400001 | 552 | 000639037800024 | 712 | 000551233000037 | 872 | 000165549500017 | 1032 | 000354012100012 | 1192 | 000084158500004 |
| 73 | A1996TP22200021 | 233 | 000428260000005 | 393 | A1997YH25500009 | 553 | 000405594900006 | 713 | 000075155200002 | 873 | 000604402000007 | 1033 | 000183300400024 | 1193 | 000081511100011 |
| 74 | A1997XL76900003 | 234 | 000370157900003 | 394 | A1995RW87900025 | 554 | 001079761200001 | 714 | 000309090200012 | 874 | 000256719300009 | 1034 | 000637015600021 | 1194 | 000776768900001 |
| 75 | 000567896600001 | 235 | 000346976700045 | 395 | A1997WX98700045 | 555 | 000365370500015 | 715 | 000309090200007 | 875 | 000697619800001 | 1035 | 000604402000022 | 1195 | 000234698100012 |
| 76 | 000389238700019 | 236 | 000607577600004 | 396 | 000249999400003 | 556 | 000353602500018 | 716 | 001087241100004 | 876 | 000373797100007 | 1036 | 000251037000007 | 1196 | 000244793000001 |
| 77 | 000562766500001 | 237 | 000533615600001 | 397 | 000674223800003 | 557 | 000402496400003 | 717 | 000175197000001 | 877 | 000579368400002 | 1037 | 000087469200008 | 1197 | 000456082500008 |
| 78 | 000511366200001 | 238 | 000343964000028 | 398 | 000309714600008 | 558 | 000397073900002 | 718 | 000809103300001 | 878 | 000864478100001 | 1038 | 000654245300002 | 1198 | 000370884100011 |
| 79 | 000183278500008 | 239 | 001112713100001 | 399 | 000246880100010 | 559 | 000415008300008 | 719 | 000469445600005 | 879 | 000348732100045 | 1039 | 000496470000008 | 1199 | 001209550100001 |
| 80 | 000269034400003 | 240 | 000311390800007 | 400 | 000728178100005 | 560 | 000697905800001 | 720 | 000877380000001 | 880 | 000330748100004 | 1040 | 000535829300002 | 1200 | 000832910200002 |
| 81 | 000257123600011 | 241 | 000309590900009 | 401 | 000275707000016 | 561 | 000327180700054 | 721 | 000461343000004 | 881 | 000668533300001 | 1041 | 000385942800003 | 1201 | 000445120700011 |
| 82 | 000401614900047 | 242 | 000083539000003 | 402 | 000289266100014 | 562 | 000171656400007 | 722 | A1992JR68900008 | 882 | 000253841300010 | 1042 | 000272298300001 | 1202 | 000893014400001 |
| 83 | 000424098900008 | 243 | A1996TX17800016 | 403 | 000084428300020 | 563 | 000259943600017 | 723 | 000253977200005 | 883 | 000168646500022 | 1043 | 000320855600002 | 1203 | 000614038400013 |
| 84 | 000397073900006 | 244 | 000850242500001 | 404 | 000475743900024 | 564 | 000083494200003 | 724 | 000171969600002 | 884 | 000238943800006 | 1044 | 000283841200002 | 1204 | 000369378900001 |
| 85 | 000250634600011 | 245 | 000396643300037 | 405 | 000492689700006 | 565 | 000082958400004 | 725 | 000227196500003 | 885 | 000187224700013 | 1045 | A1993LY46700026 | 1205 | 000604402000011 |
| 86 | 000277282700002 | 246 | 000447650100057 | 406 | 000418316100002 | 566 | A1995RU49800005 | 726 | 000166259600010 | 886 | 000232542000025 | 1046 | A1994NK92500018 | 1206 | 000467282200008 |
| 87 | 000446802800003 | 247 | A1994NG71900003 | 407 | 000760863800011 | 567 | 000079086700011 | 727 | 000265305500028 | 887 | 000261680600007 | 1047 | 000711947800007 | 1207 | 000313935800011 |
| 88 | A1996UA02400009 | 248 | A1995RP43200006 | 408 | 000641445400022 | 568 | 000077464600008 | 728 | 000459390100002 | 888 | 000539283900003 | 1048 | 000234262600019 | 1208 | 000405934700012 |
| 89 | 000308646900005 | 249 | 000472579100005 | 409 | 000344143700001 | 569 | A1996UJ22500012 | 729 | 000310123000010 | 889 | 000507461600009 | 1049 | 000247686900001 | 1209 | 000287910200006 |
| 90 | 000251840300010 | 250 | 000274905400002 | 410 | 000080624500004 | 570 | 000831977900004 | 730 | 000291175900007 | 890 | 000453337500008 | 1050 | 000249521200012 | 1210 | 000304809200004 |
| 91 | 000336898100004 | 251 | A1992JF47000002 | 411 | 000338094200021 | 571 | 000312242800003 | 731 | 000378563400004 | 891 | 000223639200004 | 1051 | 000180566700009 | 1211 | 000318971100001 |
| 92 | 001005743800001 | 252 | 000853268800003 | 412 | 000280894600050 | 572 | 000397089700028 | 732 | 000387525800004 | 892 | 000561333100002 | 1052 | 000177669300004 | 1212 | 000252677700004 |
| 93 | 000826483700002 | 253 | 000508660100017 | 413 | 001186774400001 | 573 | 001160750400081 | 733 | 000302796200059 | 893 | 000401314100051 | 1053 | 000609054400008 | 1213 | 000484118700055 |
| 94 | 000265145600021 | 254 | 000604253700001 | 414 | 000278759900034 | 574 | A1996TQ19500002 | 734 | A1995QR27900008 | 894 | 000228636600001 | 1054 | 000525755600005 | 1214 | 000407198800013 |
| 95 | 000258625800010 | 255 | 000517348200006 | 415 | A1996WE61500002 | 575 | A1996UJ34100057 | 735 | 000329952900001 | 895 | 000308011800010 | 1055 | 001183279200001 | 1215 | 000921354700001 |
| 96 | 000236362600047 | 256 | A1996UU50100002 | 416 | A1994PC92000021 | 576 | 000285710400009 | 736 | 000175197000002 | 896 | 000334295600009 | 1056 | 000316610700067 | 1216 | 000910240500001 |
| 97 | 000504487900014 | 257 | 000404246900029 | 417 | 000733052600005 | 577 | 000655475500002 | 737 | 000185603100001 | 897 | 000320188700008 | 1057 | 000486134700002 | 1217 | 000931161700001 |
| 98 | 000745654200002 | 258 | 000506806500015 | 418 | 000263072500013 | 578 | 000238943800004 | 738 | 000477878400009 | 898 | 000469342500015 | 1058 | 000244053600007 | 1218 | 000951309800001 |
| 99 | 001061229300001 | 259 | 001004630700001 | 419 | 000274388000012 | 579 | 000290818400007 | 739 | 000085399300006 | 899 | 000452489300006 | 1059 | 000253675900010 | 1219 | A1993LU25500006 |
| 100 | 000346393500006 | 260 | 000331782900001 | 420 | 000328417600002 | 580 | 000428093900114 | 740 | 000735993400001 | 900 | 000376091400004 | 1060 | 000223943500036 | 1220 | 000357906600010 |
| 101 | 001074548900001 | 261 | 000882171000002 | 421 | 000791331200001 | 581 | 000760863800013 | 741 | 000286156700006 | 901 | 000401497300011 | 1061 | 000228636600022 | 1221 | 000390737200018 |
| 102 | 000810036900036 | 262 | 000602274700001 | 422 | 001026664100001 | 582 | 000906713800011 | 742 | 000416496000009 | 902 | 001014573000001 | 1062 | 000693937400036 | 1222 | 001165022800008 |
| 103 | 000354897600005 | 263 | 000234154000003 | 423 | 000314357700021 | 583 | 000906713800008 | 743 | 000257513800003 | 903 | 000466389900004 | 1063 | 000275707000014 | 1223 | 000305238200017 |
| 104 | 000493944800023 | 264 | 000298358000013 | 424 | 000283621800041 | 584 | 000297118800011 | 744 | 000250683000007 | 904 | 000255767600004 | 1064 | 000272264700010 | 1224 | 000357411100015 |
| 105 | 000222909500013 | 265 | 000317164200005 | 425 | 000255555100034 | 585 | 000379629600009 | 745 | 000370393500007 | 905 | 001193673700011 | 1065 | 000267388300003 | 1225 | 000278751900016 |
| 106 | 000188665700007 | 266 | 000697475600001 | 426 | A1995RF53900002 | 586 | 000353602500012 | 746 | 000470638700001 | 906 | 000670569000001 | 1066 | 000301633900004 | 1226 | 000569706200001 |
| 107 | 000181797000004 | 267 | 000331267200019 | 427 | 000230168000004 | 587 | 000299220600025 | 747 | 000348588000013 | 907 | 000224507700011 | 1067 | 000583846900007 | 1227 | 000829796900003 |
| 108 | 000223639200001 | 268 | 000305458800009 | 428 | 000360823100030 | 588 | 000243015700015 | 748 | 000393169500012 | 908 | 000170129400004 | 1068 | 000513297000006 | 1228 | 000428533500001 |
| 109 | 000305912500025 | 269 | 000987286600011 | 429 | 001065493600001 | 589 | 000358531900007 | 749 | 000225480900024 | 909 | A1994NR56800013 | 1069 | 000076741400006 | 1229 | 000908273500001 |
| 110 | 000361813200006 | 270 | 000581034900004 | 430 | 000344870400008 | 590 | 000442189900033 | 750 | 000359741800002 | 910 | 000409341700014 | 1070 | 000798064600004 | 1230 | 001109762400003 |
| 111 | 000275927200006 | 271 | A1995RF54100003 | 431 | 000171511600024 | 591 | 000282693400008 | 751 | 000304809200008 | 911 | 000278648200009 | 1071 | 000283947200005 | 1231 | 000520867300014 |
| 112 | 000251365400013 | 272 | A1996UF61900019 | 432 | 000511318200001 | 592 | 000396943600016 | 752 | 000258580000008 | 912 | 000386087000003 | 1072 | 000483522400002 | 1232 | 000394350700005 |
| 113 | 000086947900037 | 273 | A1994PX26200006 | 433 | 000260660300011 | 593 | 000223952700007 | 753 | A1994PU67300004 | 913 | 000831977900002 | 1073 | 000293324100018 | 1233 | 000184311100005 |
| 114 | 000176867600006 | 274 | 000261513400060 | 434 | 001038200700001 | 594 | 000071151000008 | 754 | 000171721200004 | 914 | 000311838300011 | 1074 | 000301031700008 | 1234 | 000245971800004 |
| 115 | A1997WG56400009 | 275 | 000402496400007 | 435 | 000165497300009 | 595 | 000543112700001 | 755 | 000172748200005 | 915 | 000294552400001 | 1075 | 000084690000016 | 1235 | 000604597400001 |
| 116 | 000728931500012 | 276 | 000382941800004 | 436 | 000178090500009 | 596 | 000289597600007 | 756 | 000224703500033 | 916 | A1993KU08000021 | 1076 | 000370118300008 | 1236 | 000349560000003 |
| 117 | 001036824500008 | 277 | 000242471300024 | 437 | 000272987500016 | 597 | 000397073900004 | 757 | 000397985500027 | 917 | 000313562900012 | 1077 | 000701147400001 | 1237 | 001081238600001 |
| 118 | 000319107900036 | 278 | 000235905000005 | 438 | 000304580100002 | 598 | 000450375000004 | 758 | 001169536700001 | 918 | 000244793000004 | 1078 | 000346271000005 | 1238 | 000514737100001 |
| 119 | 000911481900001 | 279 | 000819552400001 | 439 | 000454091500011 | 599 | 000319727700010 | 759 | A1997XG42300003 | 919 | A1996VG13300010 | 1079 | 000415008300014 | 1239 | 000422712600010 |
| 120 | 000322402600002 | 280 | 000220255600019 | 440 | 000336873600002 | 600 | 000288060300026 | 760 | 000167443500002 | 920 | A1996UJ22500013 | 1080 | 000235893700003 | 1240 | 000767957100001 |
| 121 | 000342257400009 | 281 | 000445721800012 | 441 | 000949945800001 | 601 | 000310588500035 | 761 | 000080419400036 | 921 | 000295496100013 | 1081 | 000353212600025 | 1241 | 000555005700048 |
| 122 | 000336712900016 | 282 | 000541076700006 | 442 | 000872146300001 | 602 | 000293324100006 | 762 | 000280117800014 | 922 | 000318910000007 | 1082 | 000372580400005 | 1242 | 001109762400006 |
| 123 | 000696787400015 | 283 | 000538570900006 | 443 | 000208639700006 | 603 | 000465098600011 | 763 | 000811783000001 | 923 | 000385942800005 | 1083 | 000458713600011 | 1243 | 000328077100005 |
| 124 | 000266891500022 | 284 | 000272257700012 | 444 | 000425365100007 | 604 | 000289329500012 | 764 | 000732715700035 | 924 | 000359741800003 | 1084 | 000261517700017 | 1244 | 000423221600095 |
| 125 | A1997YH43500016 | 285 | 000518041300035 | 445 | 000295198500022 | 605 | 000349532700004 | 765 | 000924912900001 | 925 | 000544579800001 | 1085 | 000426952600007 | 1245 | 000260752000056 |
| 126 | 000803934100001 | 286 | 000402219600010 | 446 | 000243416800029 | 606 | 000278933800082 | 766 | A1995QQ15000012 | 926 | 000302601100010 | 1086 | 000640963100006 | 1246 | 000583591000001 |
| 127 | 000223639200002 | 287 | 001154421100001 | 447 | 001093360500001 | 607 | 000368009900008 | 767 | 000445721800011 | 927 | 000853127700001 | 1087 | 000545455000006 | 1247 | 000612179600001 |
| 128 | 000233504700007 | 288 | 000423176400002 | 448 | 000361889000007 | 608 | 000325495300006 | 768 | 000089958600010 | 928 | 000181229200015 | 1088 | 000249096600020 | 1248 | 000353247000020 |
| 129 | 000230444800011 | 289 | 000075506800002 | 449 | 000684140400001 | 609 | 000357086600014 | 769 | 000348430100001 | 929 | 000910947100003 | 1089 | 000285320400008 | 1249 | 000697162600002 |
| 130 | 000418038700001 | 290 | 000705596800006 | 450 | 000452539100161 | 610 | 000294417000014 | 770 | 000374369900011 | 930 | 000181853700006 | 1090 | 000239760200002 | 1250 | 000675849900008 |
| 131 | 000257758900008 | 291 | 000086843700035 | 451 | 000555865900001 | 611 | 000079129600013 | 771 | 000268607700020 | 931 | 000223249900009 | 1091 | 000288301500002 | 1251 | 001066098800001 |
| 132 | 000322290700023 | 292 | 000175118500039 | 452 | 000405361500008 | 612 | 000372603100001 | 772 | 000222444400013 | 932 | 000293324100014 | 1092 | 000316434100003 | 1252 | 000563616600001 |
| 133 | 000299753600002 | 293 | 000080761400026 | 453 | 000230868800006 | 613 | 000448901400018 | 773 | 000258921900006 | 933 | 000249791700001 | 1093 | 000254472400051 | 1253 | 000582306600008 |
| 134 | 000306178100002 | 294 | 000222393800008 | 454 | 000183872500001 | 614 | 000397551500007 | 774 | 000187284200026 | 934 | 000250671400006 | 1094 | 000316409800060 | 1254 | A1993LE63600010 |
| 135 | 000962889900001 | 295 | 000186322100002 | 455 | 000080624500008 | 615 | 000783264000001 | 775 | 000336588000005 | 935 | 000760863800014 | 1095 | 000251207600021 | 1255 | 000535764300001 |
| 136 | 000083050700005 | 296 | 000377257600028 | 456 | 000386569800002 | 616 | 000352666900008 | 776 | 000348040700057 | 936 | 000562830100016 | 1096 | 000251930800003 | 1256 | 000175709500012 |
| 137 | A1995TE65300002 | 297 | 000242308800001 | 457 | 000291169900006 | 617 | 000466389900001 | 777 | 000166803400020 | 937 | 000085028300022 | 1097 | 000252319000002 | 1257 | 000294417000038 |
| 138 | A1994PE52000004 | 298 | 000235867300007 | 458 | 000846440700001 | 618 | 000879466300001 | 778 | 000207944500001 | 938 | 000166615800012 | 1098 | 000229351000067 | 1258 | 000306308200002 |
| 139 | 000253329000002 | 299 | 000382285100023 | 459 | 000382928300007 | 619 | 001181991000001 | 779 | 000285205400004 | 939 | A1996VE44300064 | 1099 | 000249999400008 | 1259 | 000760275900002 |
| 140 | 000316434100002 | 300 | 000745657500001 | 460 | 000993395300001 | 620 | 000400017900006 | 780 | 000434963700005 | 940 | 000221265000041 | 1100 | 000086840500018 | 1260 | 000372846800002 |
| 141 | 000466622600008 | 301 | 001204532900001 | 461 | 000365831600043 | 621 | 000498052500007 | 781 | 000536286000003 | 941 | 000353602500019 | 1101 | 000266921400004 | 1261 | 000254172600013 |
| 142 | A1997XG42300004 | 302 | 000286470500029 | 462 | 000084873100041 | 622 | 000462252800001 | 782 | 000449959100002 | 942 | 000233393100076 | 1102 | 000222729500004 | 1262 | 000261169800009 |
| 143 | 000222729500005 | 303 | 000267220600030 | 463 | 000271779600011 | 623 | 000303299900061 | 783 | 000088461000007 | 943 | 000305458800002 | 1103 | 000226821700009 | 1263 | 000523247500002 |
| 144 | 000244287100021 | 304 | 000298620200022 | 464 | A1996VD63700001 | 624 | 000275571100005 | 784 | 000413707600003 | 944 | 000796545300004 | 1104 | 000181853700007 | 1264 | 000261636900009 |
| 145 | 000184965800011 | 305 | 000281459800008 | 465 | 000375173200002 | 625 | 000330205800001 | 785 | 000405181200014 | 945 | 000929556800001 | 1105 | 000336483400006 | 1265 | 000761050800001 |
| 146 | 000185020800001 | 306 | 000183768600019 | 466 | 000240433000004 | 626 | 000321853600010 | 786 | 000434933400057 | 946 | 000267225600007 | 1106 | A1996VD88000045 | 1266 | 000460514700001 |
| 147 | A1991FN35200008 | 307 | 000275965100009 | 467 | 000654657600009 | 627 | 000320093100015 | 787 | 000901175800001 | 947 | 000230203300008 | 1107 | 000072654000015 | 1267 | 000438714500011 |
| 148 | 000172873300022 | 308 | 000174769300020 | 468 | 000260752000042 | 628 | 000627850200001 | 788 | 000080433900001 | 948 | 000241112400013 | 1108 | 000080426400011 | 1268 | 000413167500037 |
| 149 | 000299610200034 | 309 | 000296771800001 | 469 | 000279610700003 | 629 | 000570522400001 | 789 | 000859948800037 | 949 | 000312630000005 | 1109 | 000450048400049 | 1269 | 000562335400003 |
| 150 | A1997WG56400003 | 310 | 000245754300033 | 470 | 000302591400003 | 630 | 000307192100013 | 790 | 000689227200001 | 950 | 000616574600001 | 1110 | 000394388300007 | 1270 | 000351191400012 |
| 151 | 000370911900004 | 311 | 000251145000007 | 471 | 000398740600002 | 631 | 001068054300001 | 791 | 000281292100009 | 951 | 000568330400001 | 1111 | 000352203200003 | 1271 | 000843542400002 |
| 152 | 000170634600010 | 312 | 000086732100005 | 472 | 000518629700001 | 632 | 000550003300001 | 792 | 000387266900007 | 952 | 001058417800001 | 1112 | 000171475200008 | 1272 | 000942716000001 |
| 153 | A1997WX37500010 | 313 | 000346930200009 | 473 | 000711692800008 | 633 | 000664510200001 | 793 | 000286540100004 | 953 | 000379742800007 | 1113 | 000655916400001 | 1273 | 000374724000008 |
| 154 | 000274537400015 | 314 | 000522648000002 | 474 | 000635699600031 | 634 | 000411879000001 | 794 | 000272065300011 | 954 | 000083060000011 | 1114 | A1995TT19500014 | 1274 | 000424400000157 |
| 155 | 000177107900007 | 315 | 000179888100006 | 475 | 000412996900001 | 635 | 000371996300008 | 795 | 000228135100002 | 955 | A1996VP22600023 | 1115 | 000307440300006 | 1275 | 000175437200023 |
| 156 | 000363058900017 | 316 | 000310550500008 | 476 | 000390621400004 | 636 | 001188166400001 | 796 | 000168646500007 | 956 | 000337659400007 | 1116 | 000298281100011 |  |  |
| 157 | 001012152700001 | 317 | 000389635300035 | 477 | 000783162800001 | 637 | 000512886200034 | 797 | 000463206800021 | 957 | 000661584100001 | 1117 | 001066078100001 |  |  |
| 158 | 000258013800022 | 318 | 000405361500007 | 478 | 000084829000004 | 638 | 000408228000001 | 798 | 000664711400001 | 958 | 000888905100027 | 1118 | 000569728400010 |  |  |
| 159 | 000664254900001 | 319 | 000338540300008 | 479 | 000345023400050 | 639 | 000345643900006 | 799 | 000079405500023 | 959 | 000252563600001 | 1119 | 000225755800005 |  |  |
| **160** | **000531609300001** | **320** | **000358531900003** | **480** | **000761394100026** | **640** | **000366181600001** | **800** | **000357769400008** | **960** | **000220485800007** | **1120** | **000240928600030** |  |  |
